# Tools to manage the decision-making process in operating rooms

**DOI:** 10.1186/1472-6963-14-S2-P86

**Published:** 2014-07-07

**Authors:** Liliana Neriz, Daniela Silva, Francisco Ramis, Alicia Núñez

**Affiliations:** 1 Department of Management Control and Information Systems, School of Economics and Business, Universidad de Chile, Santiago, Chile; 2 Department of Industrial Engineering, Center of Advanced Studies in Process Simulation, Universidad del Bío-Bío, Concepción, Chile

## Background

This study aims to build tools to manage and support decision making processes in operating rooms and related units. Activity-based costing (ABC) was used in complement with performance measurement tools. The tools constructed were validated in five different hospitals in Chile; in this study we present the results from one of these hospitals.

## Methods

We analyzed and created a model to deal with and better manage current problems in operating rooms such as inefficiency in the use of resources, low productivity, extended waiting times, and the elevated costs associated with providing surgical services. By gathering data from the surgical process, we built a dictionary of activities, identified the cost-drivers and the cost object to trace the overhead cost using the ABC methodology. In combination with the information obtained we identified indicators to measure performance associated to operating room use, using @risk software to simulate the optimum performance for continuous improvement.

## Results

Based on the application of the data we found a disparity between the actual hospital costs and public health care insurance coverage suggesting a need to improve operating room efficiency to achieve sustainability. We also found gaps between the observed and ideal performance indicators and noted that time is a major factor.

## Conclusions

The results of this study shows that implementing ABC and performance measurement tools lead to operational improvements and better strategic decisions about rationalizing services, and improve hospital self-management.

